# Effect of Decellularized Extracellular Matrix Bioscaffolds Derived from Fibroblasts on Skin Wound Healing and Remodeling

**DOI:** 10.3389/fbioe.2022.865545

**Published:** 2022-06-29

**Authors:** Hyo-Sung Kim, Hyun-Jeong Hwang, Han-Jun Kim, Yeji Choi, Daehyung Lee, Hong-Hee Jung, Sun Hee Do

**Affiliations:** ^1^ Department of Veterinary Clinical Pathology, College of Veterinary Medicine, Konkuk University, Seoul, South Korea; ^2^ Terasaki Institute for Biomedical Innovation, Los Angeles, CA, United States; ^3^ Advanced Medical Device R&D Center, HansBiomed Co. Ltd., Seoul, South Korea

**Keywords:** fibroblast, decellularized extracellular matrix (dECM), biomaterial, skin wound, remodeling, diabetic ulcer

## Abstract

The mammalian tissue extracellular matrix (ECM) has been used as a scaffold to facilitate the repair and reconstruction of numerous tissues. However, the material properties of decellularized ECM (dECM) from *in vitro* cell cultures and the effect of these properties on wound remodeling remain unclear. To elucidate its biological activity, we extracted dECM from human lung fibroblasts, fabricated it into a patch, and applied it to a full-thickness skin wound. The fibroblast-derived dECM (fdECM) maintained the content of collagen Ⅰ, collagen Ⅳ, and elastin, and the extraction process did not damage its critical growth factors. The fdECM-conjugated collagen patch (COL-fdECM) facilitated wound contraction and angiogenesis in the proliferative phase when applied to the *in vivo* full-thickness skin wound model. Moreover, the COL-fdECM treated wound showed increased regeneration of the epidermal barrier function, mature collagen, hair follicle, and subepidermal nerve plexus, suggesting qualitative skin remodeling. This therapeutic efficacy was similarly observed when applied to the diabetic ulcer model. fdECM was shown to help remodel the tissue by regulating fibroblast growth factors, matrix metalloproteinases, and tissue inhibitors of metalloproteinases via the p38 and ERK signaling pathways in an *in vitro* experiment for understanding the underlying mechanism. These results provide a biological basis for cell-derived ECM as a multi-functional biomaterial applicable to various diseases.

## Introduction

The decellularized xenogeneic scaffolds using various tissue types such as small intestine submucosa, cartilage, heart valves, and urethra have generated considerable interest in tissue engineering to develop functional bioscaffolds as natural templates (Cui et al., 2019). Although the standard procedure has not been established, the basic principle is to remove cells from tissues or organs using appropriate methods, leaving the bioactive component (Massaro et al., 2021). This organ-derived extracellular matrix (ECM) preserves its natural 3D structure and multidirectional active composition, leaving critical biocompatibility, immunogenicity, cell penetrability, and disease transmission issues. However, cell-derived ECM containing native bioactive molecules and proteins overcome these organ-derived ECM disadvantages by avoiding pathogen risks and immunogenic molecules (Lu et al., 2011b). Therefore, ECM extraction from *in vitro* cell culture has recently received attention as an alternative to extracting ECM from tissues or organs. This approach allows for the gathering of a sufficient amount of well-characterized ECM and controlling the active ingredient content to support *in vivo* function. Moreover, *in vitro* generated ECM has the advantages of a physiological system with standardizable and customizable modifications toward a specific application. Although 2D-cultured cell-derived ECM lacks 3D architectures, technological advances have supplemented the cell-derived ECM structural limitations by integrating it with other materials, cell aligning, and 3D culture (Kim et al., 2015; Assuncao et al., 2020; Antich et al., 2021). Furthermore, cell-derived ECM may be utilized for autologous ECM preparation and can be combined with different cell-derived ECM types to maximize efficacy (Lu et al., 2011b). However, ECM bioactive functions and mechanisms remain unclear, requiring further elucidation.

In a broad sense, ECM is a union of various materials such as fibrous proteins (collagens), glycoproteins, enzymes, and growth factors. These ECM components are synthesized mainly by fibroblasts that exist most abundantly in connective tissue. The ECM in the connective tissue forms the backbone of the polymer network, providing the tissue with morphology/stability and tensile strength (Assuncao et al., 2020). Therefore, various fibroblasts have been investigated for tissue engineering and biomaterials (Lu et al., 2011a; Bourget et al., 2012). *In vitro* fibroblasts grow for 14 weeks to form thick, multilayered cell sheets and secrete abundant ECM proteins and proteoglycans (Xing et al., 2015). Decellularized ECM (dECM) derived from fibroblast sheets is composed primarily of collagen and is more like natural tissue than conventional biopolymers used in tissue engineering (Assuncao et al., 2020). In addition to a supportive role, previous studies have shown that cell-derived ECM can significantly improve cell adhesion, migration, and proliferation to acquire morphology similar to that of the original tissue (Lu et al., 2011b; Lin et al., 2012). Specifically, fibroblast-derived dECM (fdECM) helps keratinocytes proliferate by maintaining their stem-like state and aiding peripheral nerve regeneration by controlling the neurite outgrowth (Harris et al., 2017; Wong et al., 2019). In addition, fdECM plays a role in angiogenesis by aiding endothelial cell sprouting and lumen formation (Newman et al., 2011). In particular, WI-38 lung fdECM has been reported to be rich in angiogenic factors that help skin wound healing (Du et al., 2020). Moreover, it has been suggested that fdECM help recruit macrophages and/or modulate macrophage phenotype through *in vivo* study (Savitri et al., 2020).

Researchers have attempted to develop biomaterials using these cell-derived ECM characteristics for the regeneration of various tissues, such as blood vessels, tendons, muscles, bones, and cartilages (Lu et al., 2011b; Gu et al., 2014; Kim et al., 2015; Xing et al., 2017; He et al., 2021). However, the integumentary system also needs such tissue engineering to treat wide and intractable skin defects such as burns, pressure ulcers, and diabetic ulcers. Therefore, this study was designed to evaluate the ECM bioactive factors obtained from the well-known, WI-38 human lung fibroblast cultures and investigate the effect of fdECM in chronic wound healing for future applications. Moreover, we elucidated the molecular biological mechanisms underlying the therapeutic efficacy of fdECM, which is yet to be fully understood despite recent advances and efforts in ECM technology.

## Materials and Methods

### Preparation of Decellularized Extracellular Matrix

Human lung fibroblast WI-38 (CCL-75, ATCC) were seeded at 2 × 10^4^ cells/cm^2^ into a T-175 flask and cultured for five to seven days until confluence (approx. 9 × 10^4^ cells/cm^2^) was reached in Dulbecco's Modified Eagle medium (DMEM; #11995-065, Gibco) supplemented with 10% fetal bovine serum (FBS; Gibco) and 1% antibiotics (#15140122, Gibco). The medium was changed every 3 days. After reaching 100% confluency, the cells were washed twice with Dulbecco’s phosphate-buffered saline (DPBS; Sigma-Aldrich) and lysed with a 0.25% Triton X-100 solution (#H5141, Promega) containing 10 mM NH_4_OH (#221228, Sigma-Aldrich) for 90 s at 37°C. After the decellularization process, 50 U/mL Deoxyribonuclease I (#18047-019, Invitrogen) and RNase A (#12091-039, Invitrogen) was added to the lysate for 90 min, and the resulting solution was frozen overnight in 50 mL conical tubes at -80°C and lyophilized.

### Componential Analysis of Decellularized Extracellular Matrix

For western blot analysis, dECM was washed with PBS and then scraped to collect the sample. The sample was extracted from RIPA buffer containing a protein inhibitor cocktail (#89900, Thermo Fisher Scientific). The total protein quantity before and after decellularization was measured by bicinchoninic acid assay (#23225, Thermo Fisher Scientific). Samples (20 µg) were separated by electrophoresis of a 7.5% polyacrylamide gradient gel, and the separated proteins were transferred to a nitrocellulose membrane (HATF29325, Millipore). The membrane was blocked with 2.5% bovine serum albumin (BSA) in Tris-buffered saline (TBS), and primary and horseradish peroxidase-labeled secondary antibodies: anti-collagen I (1:1,000, ab138492, Abcam), -collagen III (1:1,000, ab184993, Abcam), -collagen IV (1:1,000, ab6586, Abcam), -fibronectin (1:1,000, ab6328, Abcam), -laminin (1:800, ab11575, Abcam), -elastin (1:800, ab213720, Abcam), and -β-actin (1:1,000, ab8226, Abcam) were added, incubated overnight at 4°C and proteins were detected. The membrane was washed, and the protein bands were detected using a chemiluminescence reagent (#32106, Thermo Fisher Scientific). Images were captured using the ImageQuant LAS 500 (GE Healthcare).

Relative growth factor levels were determined using a sandwich immunoassay array kit (ARY007, R&D systems). Briefly, the extracted ECM total protein concentration was assessed using a bicinchoninic acid assay, and 200 μg of total protein was used as input for the array. Chemiluminescence was detected using an ImageQuant LAS 500, and the signal intensities were measured using ImageQuant TL software (GE Healthcare).

### Fabrication of Bioactive Scaffolds

The bioactive scaffolds were fabricated using a collagen solution and ECM powder for lyophilization within the mold. Specifically, Type I porcine collagen (MS Collagen; MSBIO) was reconstituted in 0.001N HCl at 5 mg/mL at 4°C under continuous gentle magnetic stirring (COL group). After the collagen was completely dissolved, the solution was mixed with fdECM (0.1%, w/v) (COL-fdECM group). Afterward, the mixture solution either for the COL or COL-fdECM group was poured into the 12 mm diameter mold to form scaffolds, collagen patch or fdECM-supplemented collagen patch, and they were frozen for 1 day at −78°C prior to lyophilization.

### *In Vivo* Full-Thickness Skin Wound Healing Study

All experimental procedures for *in vivo* full-thickness skin wound study were approved by the Institutional Animal Care and Use Committee of Konkuk University (KU18128). Male 6-week-old C57BL/6 mice were purchased from Orient Bio. The mice were housed under controlled conditions at a temperature of 23 ± 2°C, humidity of 50 ± 5%, and a light-dark cycle of 12/12 h. To establish the full-thickness skin wound model, the surgical area was clipped and aseptically prepared using povidone iodine. The surgical excision was made on the dorsal skin of mice using a 10 mm biopsy punch under inhalation anesthesia with isoflurane (3.5% for induction and 1.5% for maintenance). The mice were randomly allocated into three groups (*n* = 5 per group), including a CON group with non-treatment, a COL group with collagen patch treatment, and a COL-fdECM group with fdECM-collagen patch treatment. After applying the allocated treatment, the skin wound was covered with Tegaderm and Coban to maintain humidity and protect from infection. Treatment was repeated for three consecutive days after the wound was induced, and the wound size was measured after 7 and 14 days. Prophylactic antibiotics (enrofloxacin, 5 mg/kg) and analgesics (meloxicam, 1 mg/kg) were administered subcutaneously after surgery and for 2 days afterward.

### *In Vivo* Diabetic Skin Ulcer Healing Study

All experimental procedures for *in vivo* diabetic skin ulcer healing study were approved by the Institutional Animal Care and Use Committee of Konkuk University (KU18042). To establish the diabetic skin ulcer model, leptin receptor knockout mice (DB), C57BL/6-Lepr^em1hwl^/Korl, were obtained from the National Institute of Food and Drug Safety Evaluation (NIFDS, Cheongju, Korea). Male 10-week-old DB and misty (wild-type) were used, and the same surgical procedure described in section *In Vivo Full-Thickness Skin Wound Healing Study* was applied. The DB mice were randomly allocated into two groups (*n* = 4 per group), the DB mice with injury-only group (DB/I) and DB mice with fdECM-collagen patch treatment group (DB/COL-fdECM). Misty mice with injury-only group (Wild/I) were used to compare the normal skin wound and diabetic ulcer.

### Histological Analysis

After 7 and 21 days after inducing the skin wound and treatment, dorsal skin samples were obtained and fixed with 10% neutral-buffered formalin (BBC Biochemical). The tissue samples were embedded in paraffin through a routine process and were sectioned at 4 µm thickness. The tissue sections were hydrated by gradient alcohols for routine histological analysis and stained with hematoxylin and eosin. In addition, tissue sections were stained with Masson’s trichrome, and specific protein expressions were visualized by immunohistochemistry for further analysis. Briefly, the hydrated tissue sections were boiled using a TintoRetriever pressure cooker (Bio SB) in sodium citrate buffer (pH 6.0) to detect α-SMA, CD34, PGP9.5, loricrin, E-cadherin, and CD31 or in Tris-EDTA buffer (pH 9.0) to detect collagen I and III. For the primary antibodies, anti-collagen I (1:200, ab6308, Abcam), -collagen III (1:200, ab7778, Abcam), -α-SMA (1:1,000, ab7817, Abcam), -CD34 (1:200, MA5-27901, Invitrogen), -PGP9.5 (1:100, ab8189, Abcam), -loricrin (1:200, ab85679, Abcam), -E-cadherin (1:100, #14472, Cell Signaling Technology) and -CD31 (1:100, sc-376764, Santa Cruz Biotechnology) were used. The anti-CD31 antibody-labeled sections were incubated with a Vectastain Elite ABC-Peroxidase kit (Vector Laboratories), and the reactions were visualized by Vector SG (Vector Laboratories). Finally, the sections were counterstained with a nuclear fast red solution (Vector Laboratories) and analyzed under a microscope (Leica Microsystems). To detect proteins other than CD31, the antibody-labeled sections were incubated with secondary antibodies conjugated with Alexa fluor 488 and/or Alexa fluor 594 (Abcam). Then the sections were mounted in 4′-6-diamidino-2-phenylindole (DAPI) containing media (H-1800, Vector Laboratories) and analyzed under a fluorescence microscope (Leica Microsystems) or confocal fluorescence microscope (Zeiss).

### *In Vitro* Wound Healing Study

Human dermal fibroblasts (PCS-201-010, ATCC) were cultured using DMEM, supplemented with 10% FBS, 1% penicillin/streptomycin (#15140122, Gibco), and 2% Mycoeraser (ME05-100, RD tech) at 37°C in a 5% CO_2_ humidified incubator. The cells were seeded on a 6-well plate at a density of 7.5 × 10^5^ cells/well and incubated for 24 h to reach the confluency. The scratch wound was made using a commercial scratcher (SPL Life Sciences). The recombinant human epidermal growth factor (rhEGF) (E9644, Sigma-Aldrich) or fdECM powder was dissolved in DPBS. The concentrated solution of rhEGF or fdECM was added to each well to a final concentration of 10 ng/mL. For the control group, the same volume of DPBS was added. Photomicrographs were taken at each time point (0, 18, 24, and 48 h) after applying the treatment. The relative scratch gap area was measured by using ImageJ software (National Institutes of Health). After 48 h when the scratch gap was restored, the cells were either fixed with 4% paraformaldehyde or the supernatant media and cells were separately harvested and stored at −78°C, for further analysis.

### Immunocytochemistry Analysis

The fixed cells were permeabilized using 0.1% Triton X-100 for immunocytochemistry analysis. The samples were blocked with 2% normal serum and incubated with anti-α-SMA (1:1000), -collagen I (1:200), and -collagen III (1:200). The signals were detected with secondary antibodies conjugated with Alexa fluor 488 or 594, and nuclei were counterstained with DAPI (D9542, Sigma-Aldrich).

### Growth Factor Array

Growth factors secreted by fibroblasts in the media were determined using a human growth factor antibody array kit (ab134002, Abcam), according to the manufacturer’s protocol. Two milliliters of supernatant media were used for each quantification. Chemiluminescence was detected using a charged-coupled device (CCD) camera (Fusion Solo S, Vilber), and the signal intensities were measured using ImageJ.

### Real-Time Polymerase Chain Reaction (qPCR)

The cells were harvested for mRNA expression analysis at -78°C. Total RNA was isolated using the RNeasy Mini kit (#74106, Qiagen) according to the manufacturer’s instructions. One microgram of extracted RNA was transcribed into cDNA using the QuantiTect Reverse Transcription Kit (#205311, Qiagen). The primers for the target genes are listed in Table 1. The PowerUp SYBR Green Master Mix (A25742, Applied Biosystems) was used to perform real-time PCR (initial denaturation, 5 min at 95°C; recurring denaturation, 15 s at 95°C, and amplification, 30 s at 60°C, for 45 cycles). All mRNA levels were normalized to mean values of reference genes, β-actin, GAPDH, 18s rRNA, β2-microglobulin, and TBP, and the results were calculated as fold changes of the cycle threshold (Ct) value relative to the controls using the 2^−ΔΔCt^ method.

**TABLE 1:** Sequences of human primers used for real-time PCR.

Target Gene	Forward Primer Sequence	Reverse Primer Sequence
FGF2	GCA​GAA​GAG​AGA​GGA​GTT​GTG	CCG​TAA​CAC​ATT​TAG​AAG​CCA​G
FGF7	TCT​TGC​AAT​GAA​CAA​GGA​AGG	CCG​TTG​TGT​GTC​CAT​TTA​GC
FGF10	CGT​ACA​GCA​TCC​TGG​AGA​TAA​C	CCC​TTC​TTG​TTC​ATG​GCT​AAG​T
MMP1	TAC​GAA​TTT​GCC​GAC​AGA​GA	AAG​CCA​AAG​GAG​CTG​TAG​ATG
MMP2	TTC​AAG​GAC​CGG​TTC​ATT​TGG	TGC​AAA​GAA​CAC​AGC​CTT​CTC
MMP3	ACT​GTG​ATC​CTG​CTT​TGT​CC	CAC​GCC​TGA​AGG​AAG​AGA​TG
TIMP1	GAT​GGA​CTC​TTG​CAC​ATC​ACT	GAT​AAA​CAG​GGA​AAC​ACT​GTG​C
TIMP2	ATC​TCA​TTG​CAG​GAA​AGG​CC	GTA​CCT​GTG​GTT​CAG​GCT​CT
TIMP3	CTT​CAC​CAA​GAT​GCC​CCA​TG	ATC​ATA​GAC​GCG​ACC​TGT​CA
β-actin	GGC​CGA​GGA​CTT​TGA​TTG​C	GTG​TGG​ACT​TGG​GAG​AGG​AC
GAPDH	ATC​AAG​AAG​GTG​GTG​AAG​CAG	GTC​GCT​GTT​GAA​GTC​AGA​GG
18s rRNA	GCC​ATG​CAT​GTC​TGA​GTA​CG	GCG​ACC​AAA​GGA​ACC​ATA​AC
β2-microglobulin	CCT​GCC​GTG​TGA​ACC​ATG​TG	TGC​GGC​ATC​TTC​AAA​CCT​CC
TBP	TGT​TGA​GTT​GCA​GGG​TGT​GG	AAA​TAA​TGC​CCC​TTC​CCG​GC

### Immunoblot Analysis

To analyze the MAPK, TGF-β/SMAD, JAK/STAT, and PI3K/AKT signaling pathways, total protein was extracted using M-PER (Thermo Fisher Scientific) extraction buffer with Halt phosphatase protease inhibitor (Thermo Fisher Scientific) according to the manufacturer’s instruction. The protein concentration was determined using a protein assay kit (Bio-Rad), and the total protein (20 µg) was separated by sodium dodecyl sulfate-polyacrylamide gel electrophoresis (SDS-PAGE) using a 4-20% precast gel (Bio-Rad). The separated proteins were transferred onto polyvinylidene fluoride membranes, followed by western blot analysis. Briefly, the background signals were blocked with EveryBlot Blocking buffer (#12010020, Bio-Rad) and incubated with each primary antibody, purchased from Cell Signaling Technology and diluted to 1:1,000 in 3% BSA/TBST (SMAD3, #9513; p-SMAD3, #9520; ERK1/2, #4695; p-ERK1/2, #4370; JNK, #9252; p-JNK, #4668; p38, #9212; p-p38, #4511; STAT3, #30835; p-STAT3, #9145; AKT, #4691; p-AKT, #4060; β-actin, #4970). After several washing steps, the membranes were incubated with horseradish peroxidase-conjugated secondary antibodies (Cell Signaling Technology). A CCD camera was used to detect immunoreactive bands using a chemiluminescent substrate (Clarity Western ECL Substrate; Bio-Rad). Quantification was performed using ImageJ, and each expression was standardized by the β-actin level.

### Statistical Analysis

Statistical analysis was conducted using Prism V5 (Graphpad Software Inc). Significant differences between groups were determined using one-way analysis of variance (ANOVA) followed by Bonferroni multiple comparisons post hoc test for parametric measures, Kruskal–Wallis test followed by Dunn post hoc test for non-parametric measures, and linear regression for construction of standard curves. Statistical significance was accepted at *p* < 0.05.

## Results

### Bioactive Component Preserved fdECM

**FIGURE 1:**
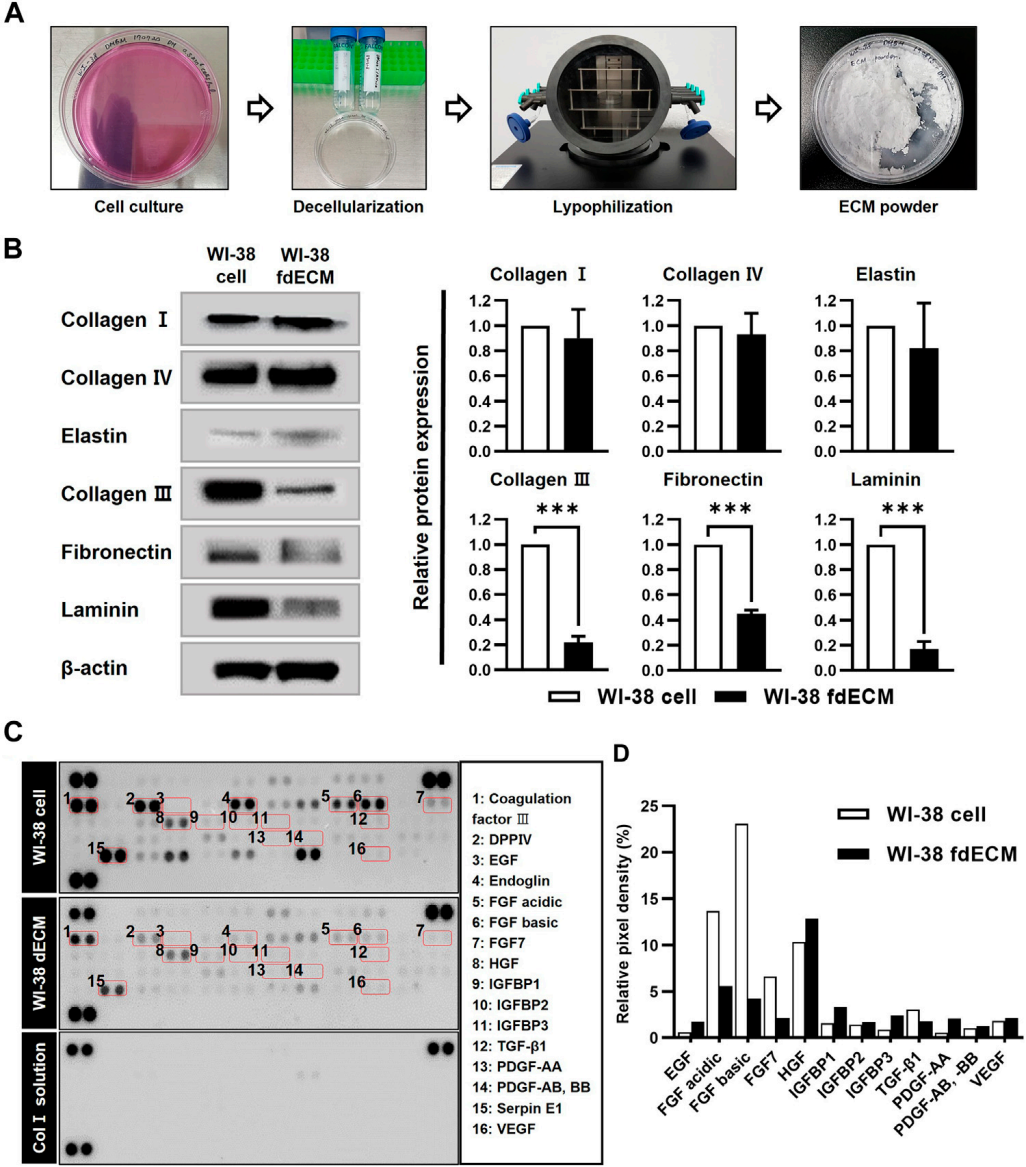
Preparation and characterization of fdECM. **(A)** The extraction method of WI-38 fibroblast-derived decellularized extracellular matrix (fdECM). **(B)** Western blot analysis of ECM proteins before and after decellularization with β-actin as an internal control. **(C)** Analysis of angiogenic factors by a semi-quantitative method. Dot blot of the matrix before and after decellularization. The graph of some of the 55 principal angiogenic factors detected (box denotes factors). Results are expressed as mean ± standard deviation (SD). ***p < 0.001.

A commercially obtained human cell line, WI-38, was used to prepare fdECM. After the cells reached full confluency, we applied an optimal decellularization protocol to remove DNA and nuclei from the cells while maximally preserving ECM content (Figure 1A and Supplementary Figure S1). The extracted ECM was approximately 100 mg per confluent T-175 flask (571 μg/cm^2^) and the ECM yield was 220 mg/g protein (*p* < 0.001, compared to extracted protein without decellularization). To verify the preservation of the active ingredients, the protein contents of cells before and after decellularization were compared by western blot. As a result, collagens I, IV, and elastin proteins showed little change, while collagen III, fibronectin, and laminin were reduced to 20, 50, and 20%, respectively, compared to the original cells (Figure 1B). In the growth factor array analysis, coagulation factor III, Serpin E1, and dipeptidyl peptidase-4 were the most abundant remaining factors even though the original contents were reduced (Figure 1C). However, several growth factors, including epidermal growth factor (EGF), hepatocyte growth factor (HGF), insulin-like growth factor-binding protein (IGFBP), platelet derived growth factor (PDGF), and vascular endothelial growth factor (VEGF) remained without being significantly lost during the decellularization process (Figure 1D).

### COL-fdECM Accelerated Skin Wound Healing *via* Neovascularization and Wound Contraction

**FIGURE 2:**
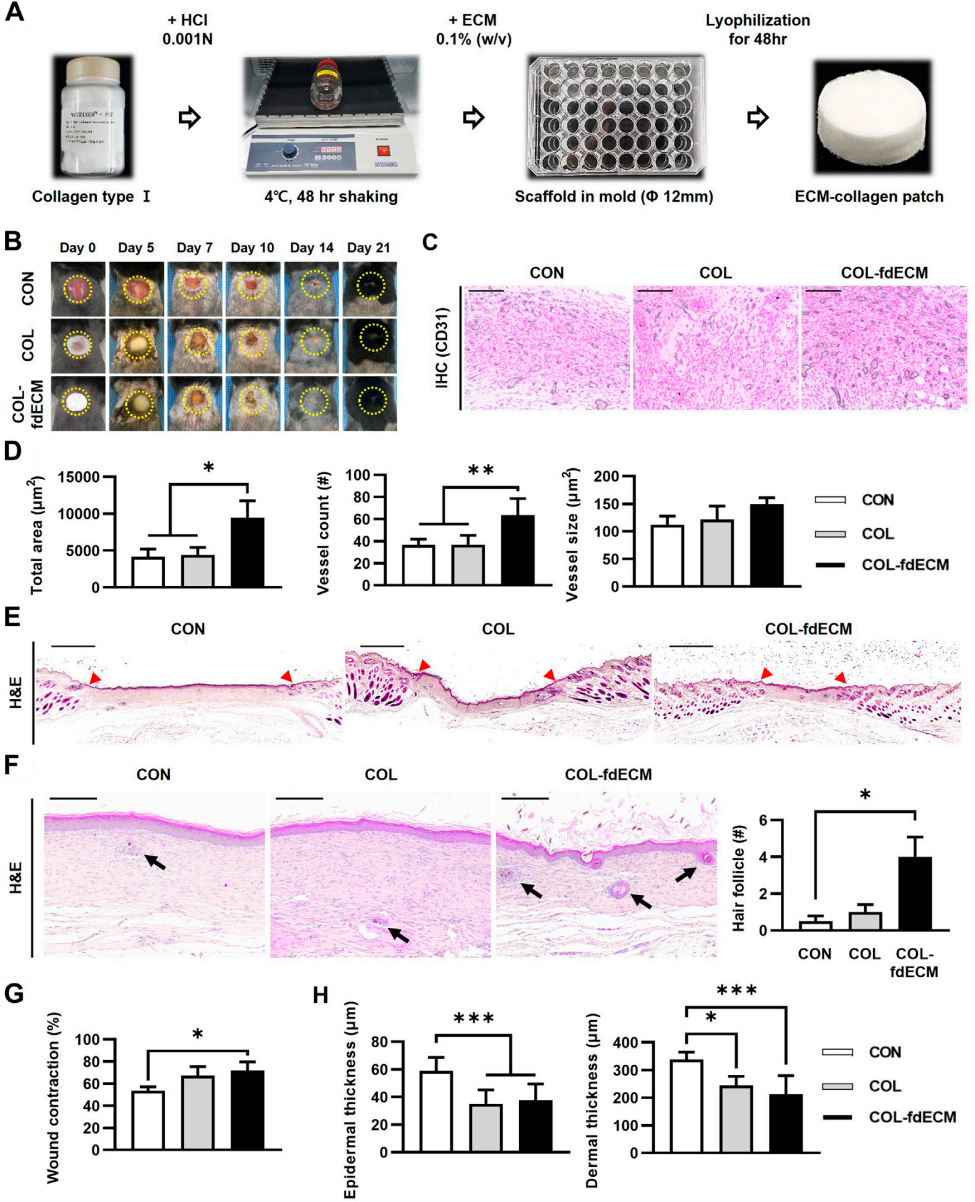
*In vivo* evaluation of fdECM scaffolds on full-thickness skin wounds. **(A)** Fabrication of fdECM scaffold by adding collagen I and lyophilization, **(B)** Gross evaluation of skin wounds, **(C)** Immunohistochemistry images stained with anti-CD31 antibody after 7 days of treatment (Gray color indicates a positive reaction), Scale bar represents 200 μm **(D)** Semi-quantitative measurement of blood vessel area, count, and size, after 7 days of treatment. **(E)** Whole-mount image of skin wound stained with hematoxylin and eosin (H&E) after 21 days of treatment. The red arrowhead indicates the initial site of the wound. Scale bar represents 2 mm **(F)** An increased number of hair follicles on regenerated dermis was detected after 21 days of fdECM treatment. Black arrows indicate hair follicles. Scale bar represents 200 μm. **(G)** Wound contraction ratio calculated as ratio of the gap between unaffected tissue to original wound diameter, **(H)** Thickness of epidermis and dermis. Bar in graphs represents the mean ± standard error of mean (SEM). *p < 0.05, **p < 0.01, ***p < 0.001.

Based on the fdECM remaining ingredients, we hypothesized that fdECM could accelerate wound healing via angiogenesis (Werner and Grose, 2003). First, we made an fdECM supplemented collagen patch to confirm its effect by adding the extracted fdECM to collagen I for the *in vivo* experiment (Figure 2A). The fdECM-collagen patch was observed to have a more homogeneous surface than the control collagen patch. However, the mechanical properties of the fdECM-collagen patch were similar to the control collagen patch, except the slightly increased compressive modulus (Supplementary Figure S2). We applied this patch to the full-thickness skin wound model to confirm its biological activities. After 14 days, in the control group (CON), where nothing was applied, the hemorrhagic crust was on the wounded skin (Figure 2B). In contrast, the defect area was almost completely epithelialized in the control collagen patch (COL) or fdECM-collagen patch (COL-fdECM) groups, and the wound area appeared smaller in the COL-fdECM group. To confirm the angiogenic efficacy of the prepared fdECM-collagen patch, we analyzed the blood vessels in the granulation tissue by CD31 immunostaining in the sampled tissue on day 7 (Figure 2C). As a result, the COL-fdECM group exhibited about twice as much blood vessel area as the other groups because the size and number of the blood vessels were significant (Figure 2D). Histological analysis was performed on the sample tissue on day 21 when the proliferation ended and remodeling was active, to confirm the long-term effect on wound healing. In the CON group, most of the defects consisted of immature skin tissue where only a few skin adnexa were observed (Figure 2E). However, in the COL-fdECM group, more hair follicles were observed in the center of the wound bed than the CON or COL group (Figure 2F). Moreover, the normal skin was closer to the center of the defect in the COL-fdECM group than the control (Figure 2G). In addition, when the collagen or fdECM-collagen patch was applied, the thickness of both the epidermis and the dermis was reduced compared to the CON group (Figure 2H). These results collectively confirm that the scaffold’s regeneration efficacy created with fdECM promoted angiogenesis and contributed to wound contraction. Furthermore, the reduced thickness of the skin and increased hair follicles in the wound bed suggest that the treatment may help wound remodeling. Therefore, we attempted additional analyzes to evaluate it.

### COL-fdECM Assisted in Remodeling the Skin Wound

**FIGURE 3:**
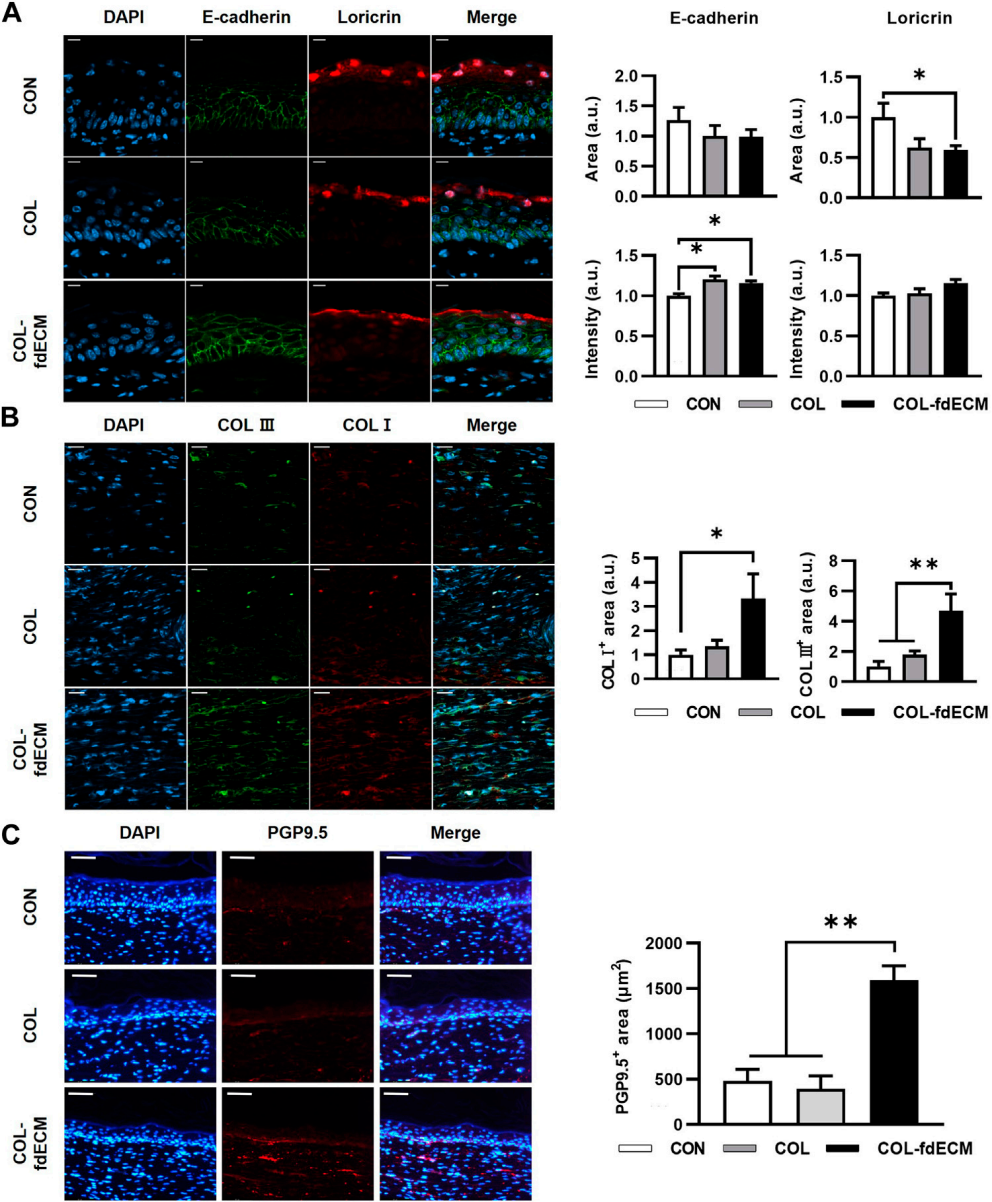
Tissue remodeling in skin wound after 21 days of fdECM treatment. **(A)** Immunofluorescence image and semi-quantitative analysis of barrier function of epidermis stained with E-cadherin (green) and loricrin (red). Scale bar represents 10 μm. **(B)** Representative images of double immunofluorescence staining against collagen I (COL I, red) and collagen III (COL III, green). Semi-quantitative analysis was performed by measuring the positive area. Scale bar represents 20 μm. **(C)** Immunofluorescence image and semi-quantitative analysis of nerve fiber in dermis stained with PGP9.5 (red). Scale bar represents 50 μm. Bar represents mean ± standard error of mean (SEM). *p < 0.05, **p < 0.01.

**FIGURE 4:**
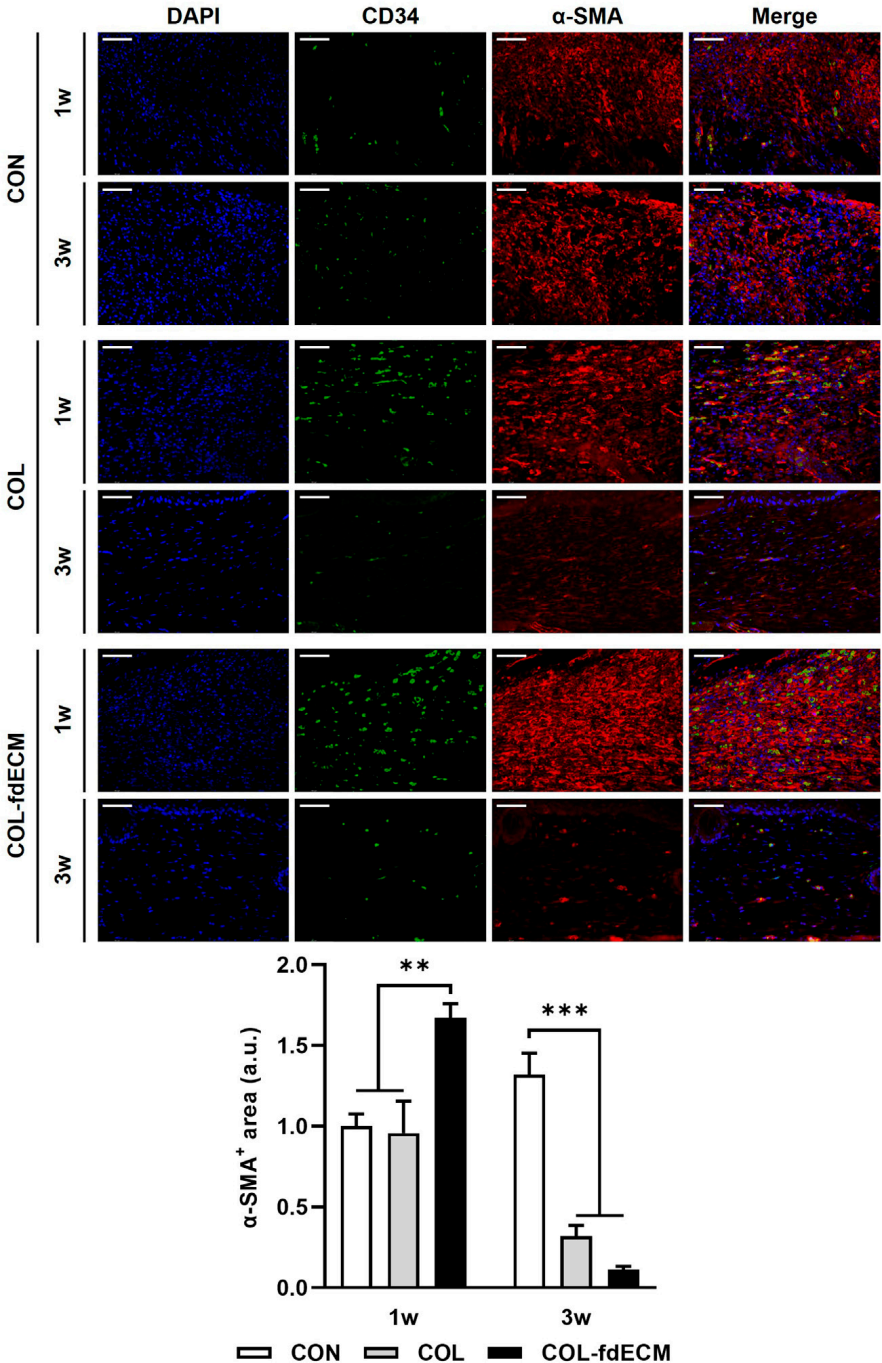
Time-varying phenotype change of dermal fibroblasts in full-thickness skin wound after fdECM treatment. Immunofluorescence image and semi-quantitative analysis of dermal fibroblast stained with CD34 (green) and α-SMA (red) on tissue sections. Scale bar represents 50 μm. Bar represents mean ± standard error of mean (SEM). **p < 0.01, ***p < 0.001.

First, we analyzed the epidermal barrier function by staining cell adhesion molecule E-cadherin and the major component of the cornified envelope, loricrin (Figure 3A) (Tunggal et al., 2005; Abhishek and Palamadai Krishnan, 2016). The E-cadherin positive area seemed larger in the control group because of epidermal thickness. However, the signal intensity was higher in both the COL and COL-fdECM groups, meaning keratinocytes adhered to each other tightly. Loricrin expression in the CON group appeared diffuse and less intense, similar to the E-cadherin expression pattern. However, in the COL-fdECM group, loricrin was expressed in a constant line. Then, we analyzed the content of major components in the dermis, collagen I and III (Figure 3B). The immunofluorescence staining confirmed that collagen I and III content in the regenerated dermis was higher in the COL-fdECM group than in the CON and COL groups. We also checked whether the cut nerve fiber re-innervated from the ganglia to the skin. The PGP9.5 positive nerve fiber area was also higher in the superficial dermis of the COL-fdECM group compared to the other groups (Figure 3C). These results indicate that the fdECM also helps remodel the skin wounds to the original tissue. To analyze the effect and mechanism of fdECM on wound remodeling, we analyzed the phenotype change of the dermal fibroblast, the cell constituting most of the skin (Figure 4). After 7 days, the α-SMA^+^ was highest in the COL-fdECM group, indicating that CD34^−^ dermal fibroblasts were mostly α-SMA^+^ myofibroblasts. Conversely, their activity was lowest at day 21 in the COL-fdECM group, suggesting dermal fibroblasts reversed their phenotype.

### fdECM Treated Fibroblasts Exhibited a Favorable State After Migration

**FIGURE 5:**
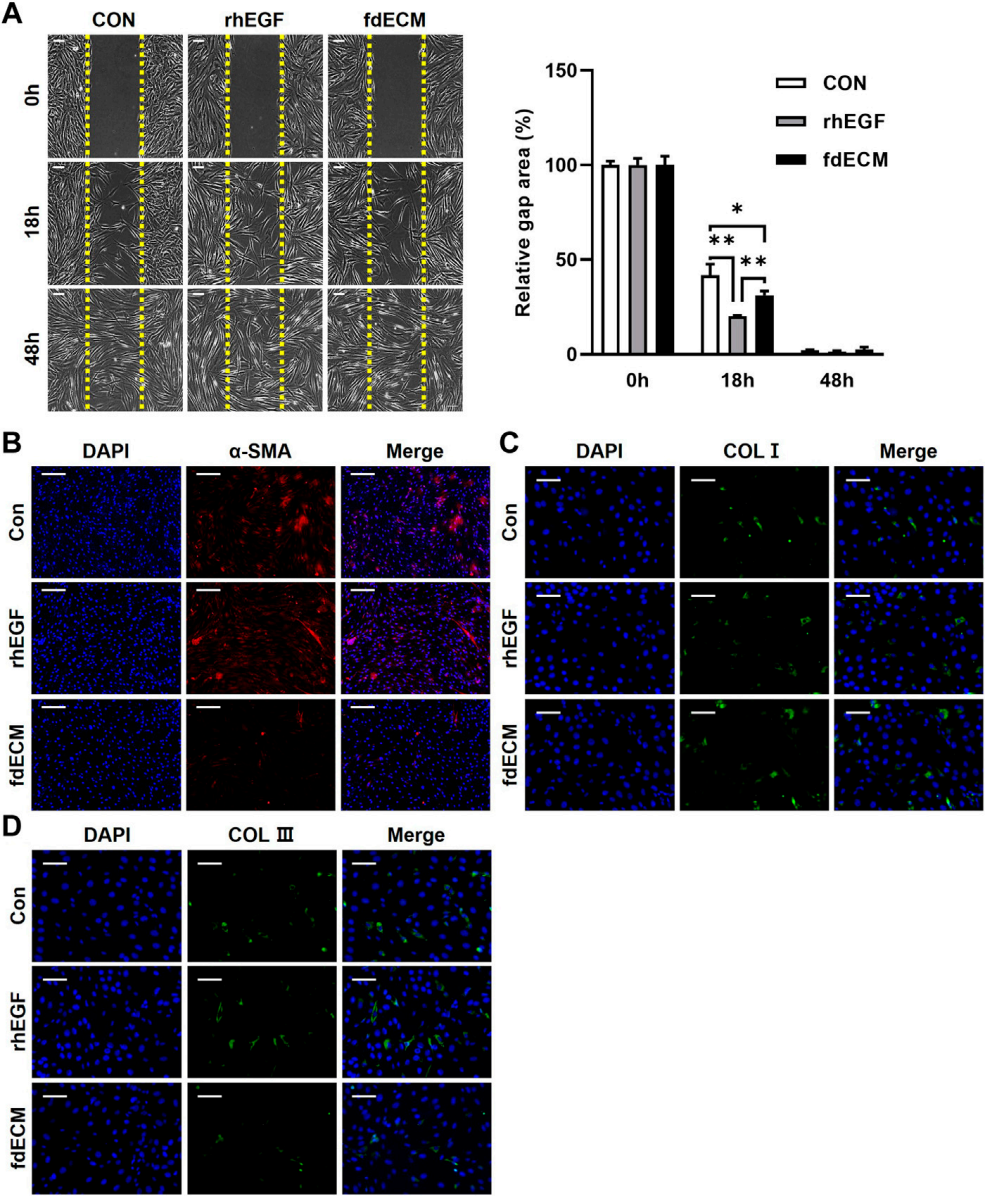
Phenotype change of human dermal fibroblasts after fdECM treatment. **(A)** Representative images of scratch wound healing assay using human dermal fibroblasts and semi-quantitative analysis of relative scratch area calculated as ratio for original defect area. Yellow dashed line indicates the original site of the scratch wound. **(B–D)** Immunofluorescence images of α-SMA **(B)**, COL I **(C)**, and COL III **(D)** expression after 48 h of fdECM treatment. Scale bars represent 200 μm **(B)** and 100 μm **(A,C,D)**. Results are expressed as mean ± standard deviation (SD). *p < 0.05, **p < 0.01.

To validate fdECM’s efficacy on fibroblasts, we treated human dermal fibroblasts with unfabricated fdECM and analyzed the behavior. As a positive control, we used rhEGF, commercially available and known for various physiological activities, including angiogenic effect. The fdECM group showed increased cell migration to the scratch gap than the control group 18 h after the scratch wound was induced, although the number of fibroblasts migrating was highest in the rhEGF group (Figure 5A). After 48 h, when the scratch wound was completely restored, the fibroblasts of the CON and rhEGF groups remained in their active form, α-SMA positive myofibroblasts (Figure 5B) (Pilcher et al., 1999). However, most fibroblasts of the fdECM group showed α-SMA negative quiescent form, consistent with the *in vivo* results. Although myofibroblasts play a major role in contracting skin wounds, the remaining myofibroblasts, after wound healing, are suspected of causing hypertrophic scar formation (Chitturi et al., 2015). Therefore, the reduced number of myofibroblasts indicates a more favorable state for wound remodeling.

**FIGURE 6:**
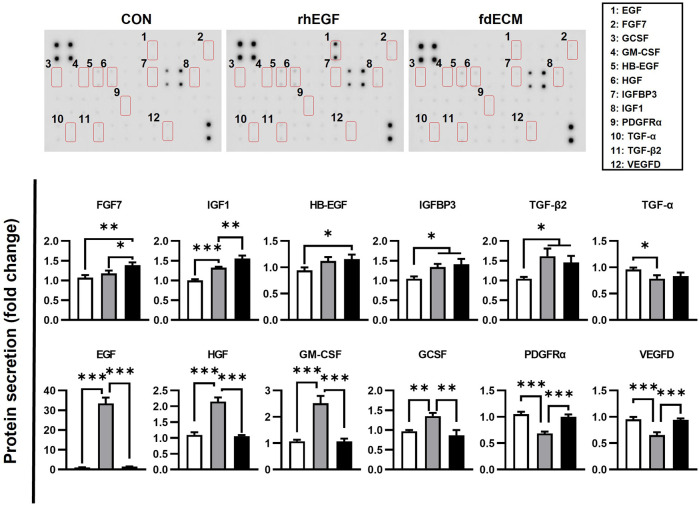
Growth factor analysis of fibroblasts after fdECM treatment. Semi-quantitative analysis of growth factors and cytokines of human dermal fibroblast after 48 h of fdECM treatment. Forty-one angiogenic factors were detected, and the significantly changed factors are graphed (box denotes factors). Results are expressed as mean ± standard deviation (SD). *p < 0.05, **p < 0.01, ***p < 0.001.

Moreover, it was confirmed that ECM components secreted by fibroblasts were different for each group. The fdECM or rhEGF treated fibroblasts were observed to secrete collagen I more than the control fibroblasts (Figure 5C). In contrast, increased collagen III-positive cells were observed in the CON and rhEGF groups and less in the fdECM group (Figure 5D). The major dermal collagen type changes from collagen III to collagen I as the skin wound is remodeled (Volk et al., 2011; Tracy et al., 2016). Therefore, these results indicate fibroblasts treated with fdECM reverse their differentiation to a stable form and secrete collagen I more than collagen III. We also evaluated fibroblasts’ growth factor secretion pattern (Figure 6). Among the 41 factors tested, fibroblast growth factor 7 (FGF7), insulin-like growth factor 1 (IGF1), insulin-like growth factor-binding protein 3 (IGFBP3), transforming growth factor beta-2 (TGF-β2), and heparin binding epidermal growth factor (HB-EGF) increased in the fdECM group compared to the CON group, but other factors did not differ. In particular, FGF7 and IGF1 were higher than in the rhEGF group, suggesting that these two factors may play a role in wound migration (Haase et al., 2003; Kirfel and Herzog, 2004).

### fdECM Modulated FGFs, MMPs, and TIMPs Expression

**FIGURE 7:**
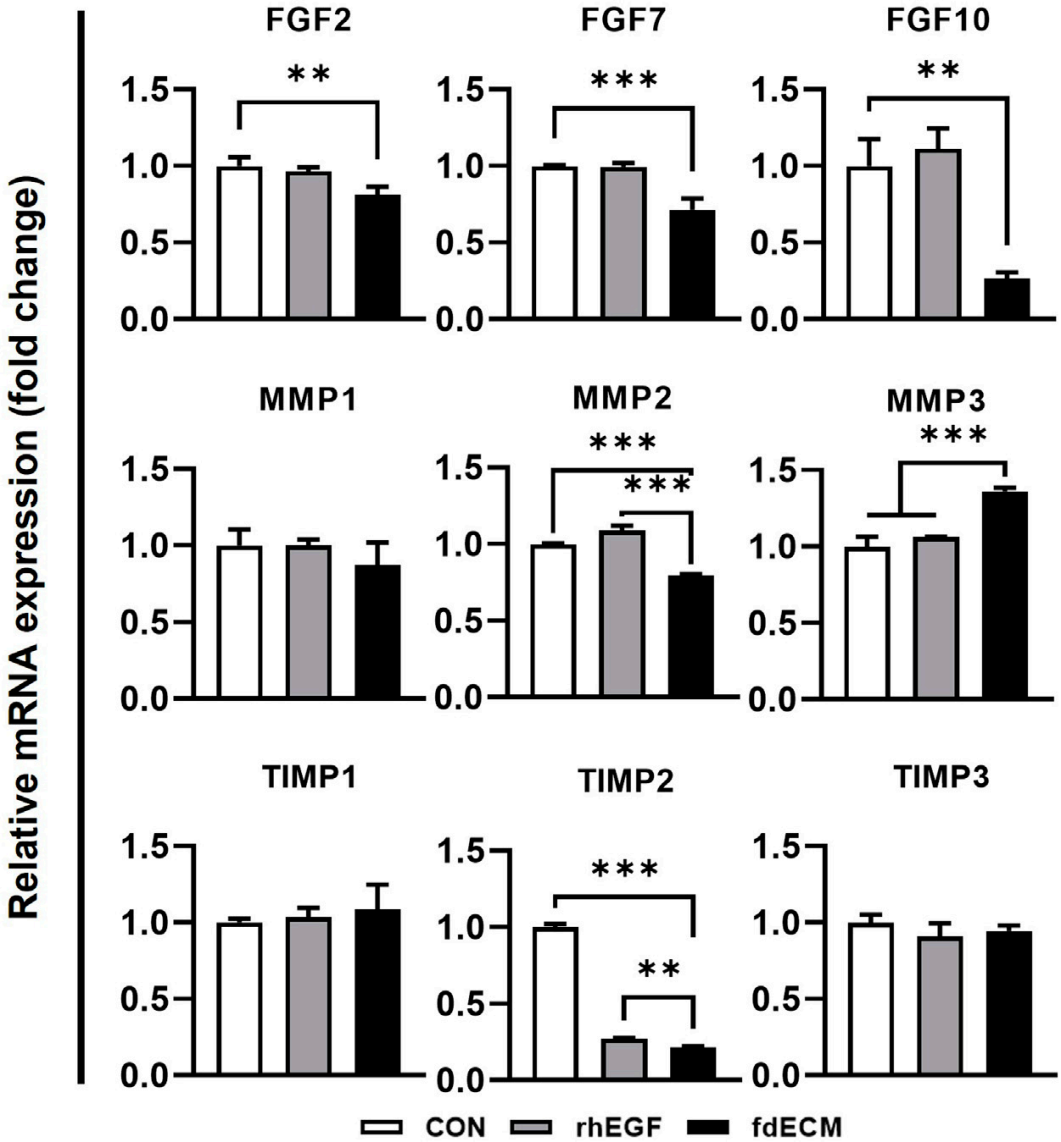
mRNA expression pattern of fibroblasts after 48 h of fdECM treatment. Real-time PCR was used to analyze mRNA expression levels after 48 h of rhEGF or fdECM treatment. Results are expressed as mean ± standard deviation (SD). **p < 0.01, ***p < 0.001.

To elucidate the underlying mechanism of fdECM on wound remodeling, mRNA expression level and cell signaling were inspected. As shown in a previous study describing that FGFs mRNA increased rapidly after wounding and then decreased as the wound healed [6], FGF-2, -7, and -10 were downregulated in the fdECM treated group after 48 h (Figure 7). Matrix metalloproteinases (MMPs) mRNA pattern after 48 h of fdECM treatment was similar to that of the late phase in skin wound healing. Proinflammatory MMPs, such as MMP1 and MMP2, were downregulated, while MMP3, remodeling MMP, was upregulated. Tissue inhibitor of metalloproteinase-2 (TIMP2) was significantly downregulated in fdECM treated dermal fibroblasts, while TIMP1 and TIMP3 were stable.

**FIGURE 8:**
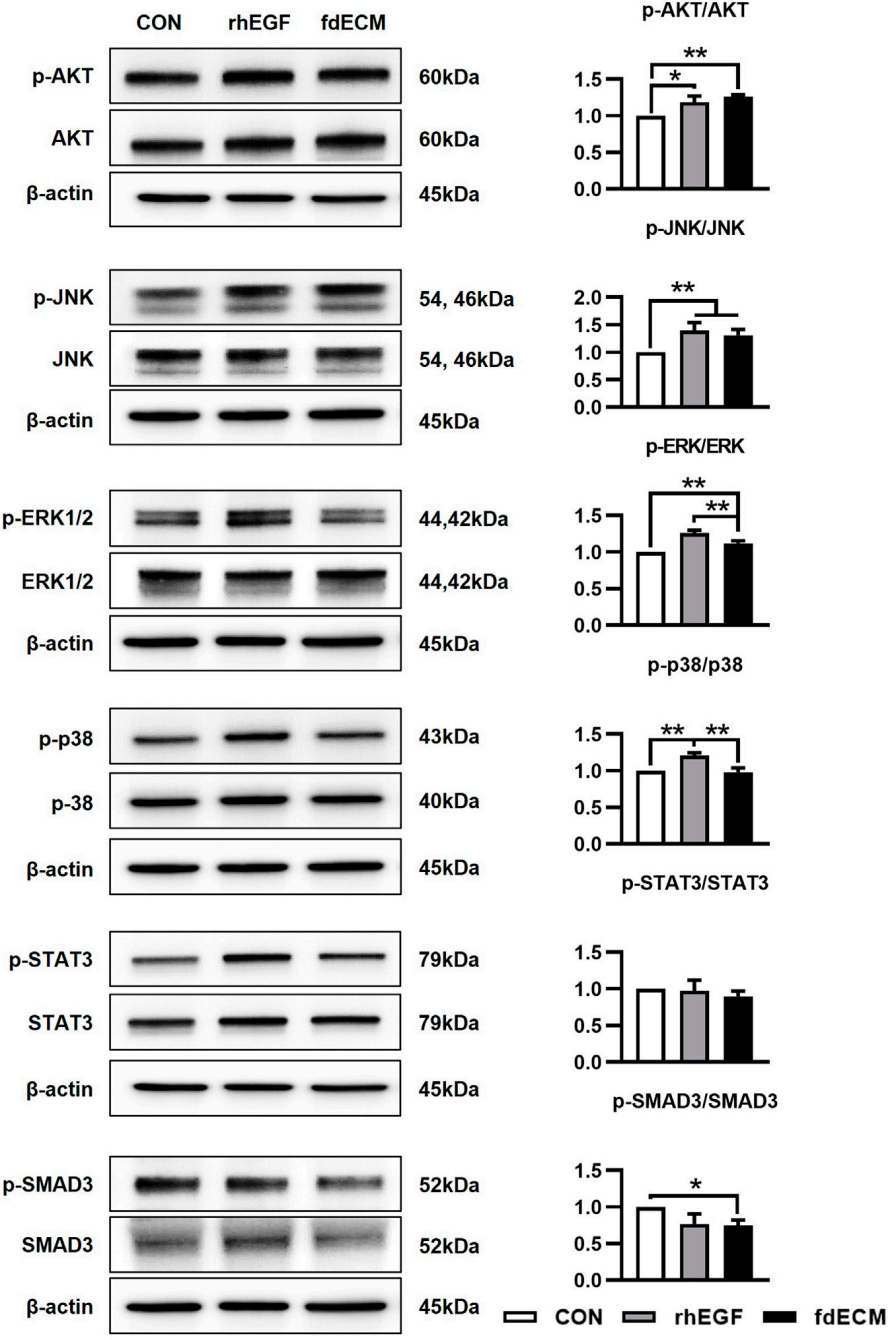
Cell signaling pattern of fibroblasts after 48 h of fdECM treatment. Western blotting was used to analyze protein expression and phosphorylation levels after 48 h of rhEGF or fdECM treatment. Results are expressed as mean ± standard deviation (SD). *p < 0.05, **p < 0.01.

We also measured the signaling pathway activated by fdECM using immunoblot analysis. Similar to rhEGF, fdECM treated fibroblasts showed increased AKT and JNK phosphorylation and decreased SMAD3 phosphorylation (Figure 8). However, the phosphorylation of ERK was decreased compared to the rhEGF group, and the p38 was decreased to the control group level. Together with the other results, these results suggest that adequate remodeling is related to regulating the p-38 and ERK pathways.

### fdECM was Effective in Diabetic Skin Ulcers

**FIGURE 9:**
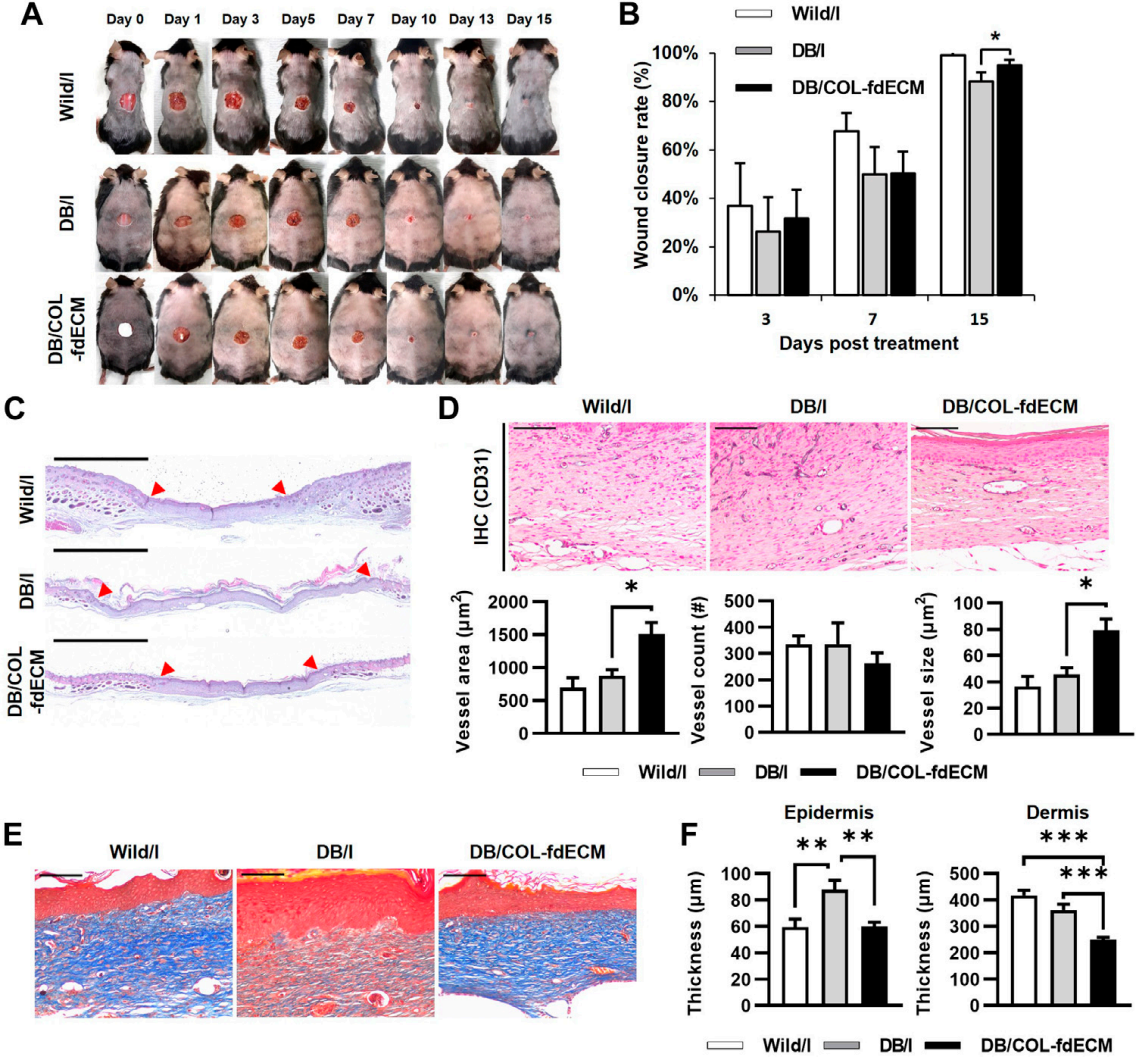
*In vivo* evaluation of fdECM scaffolds on regeneration of diabetic skin wound model. **(A)** Gross evaluation of skin wounds after 15 days of fdECM treatment, **(B)** Wound closure rate (%) measured on day 3, 7, and 15, **(C)** Whole-mount images of fdECM treated skin wound stained with hematoxylin and eosin. Red arrows indicate the original defect site. Scale bar represents 2 mm, **(D)** Immunohistochemistry images stained with anti-CD31 antibody after 15 days of treatment (Gray color indicates a positive reaction), Scale bar represents 100 μm, Vessel area, and count were calculated per dermis area (mm^2^), **(E)** Histological images of upper dermis stained with Masson’s trichrome. Scale bars represent 100 μm. **(F)** Epidermal and dermal thickness in wound area. Bar represents mean ± standard error of mean (SEM). *p < 0.05, **p < 0.01, ***p < 0.001.

Finally, we investigated whether the fdECM efficacy can also be applied to the diabetic skin wound model (Figure 9A). After 15 days of inducing the skin wound, the wound closure rate of COL-fdECM treated diabetic mice (DB/COL-fdECM) was higher than the injury-only group (DB/I) (Figure 9B). Histopathologically, the wound bed size of the diabetic ulcer model was larger compared to normal mice, and hyperkeratosis was observed (Figure 9C). However, the wound closure of diabetic ulcers was improved after applying the fdECM-collagen patch. The CD31 positive vessel area was greater in the DB/COL-fdECM group compared to the DB/I group (Figure 9D). However, the increased vessel area was due to the increased size of the blood vessels rather than the number of blood vessels. Because of the progressed wound healing stage compared to previous experiments, this result suggests that the vessels are more mature after COL-fdECM treatment. Moreover, collagen quantity in the regenerated dermis was higher after being treated with an fdECM-collagen patch, suggesting a better quality of dermal regeneration (Figure 9E). The thickness of the epidermis and dermis was also significantly reduced compared to the DB/I group (Figure 9F). These results support that fdECM and its therapeutic efficacy can be applied to chronic skin wounds such as diabetic ulcers.

## Discussion

ECM-based communication arises from orchestrating biochemical, topological, and biomechanical signals, facilitating interactive dialogue with cells that can respond through ECM remodeling. These multi-dimensional signals enable the ECM to guide complex cellular and tissue processes such as hemostasis, tissue repair, and regeneration in clinical applications. Currently, tissue- or organ-derived dECM is mainly used as a framework for tissue engineering. In addition, ECM can be used for the bare scaffolds and in various forms such as ingredients of hydrogels, polymers, nanoparticles, and in the drug delivery system (Bourget et al., 2012; Du et al., 2014; Kim et al., 2019). This study revealed the cell-derived ECM’s biological mechanism and presented the scientific basis for miscellaneous uses. Moreover, the fdECM-collagen scaffold used in this study is stable enough to be stored at room temperature for future use, making the application of regenerative medicine simple and effective.

Many attempts have been made to reveal the efficacy of ECM for tissue engineering. Particularly, the fdECM efficacy on angiogenesis is well known, although the application is different from this study (Newman et al., 2011; Aldemir Dikici et al., 2020; Du et al., 2020). However, previous research was limited in the complex evaluation of diverse tissue components or underlying mechanisms. Furthermore, studies on tissue remodeling and qualitative differences were lacking since the focus was on rapid recovery. Our results showed consistency with previous research and suggested that the effect of the ECM component itself is greater than that of the delivery system. Moreover, to link tissue engineering and biology, we aimed to comprehensively elucidate the mechanisms of proliferation to remodeling and cell signaling to protein expression. Despite such efforts, a few limitations in this study remain. First, the effect of ECM through signaling pathways has not been rigorously validated by other methods using siRNA or inhibitors. Because ECM is not a single molecule but a mixture of countless compositions, further validation methods could not be applied.

Although the skin can be easily touched and observed, it is one of the most complex organs made up of various cells. However, this study did not include all cell types constituting the skin, such as immune cells and melanocytes. However, this study presented the scientific basis for application to various other tissues and diseases by analyzing epithelial cells, fibrous tissue, nerve fibers, and blood vessels, which are typical components of tissues other than the skin. Like diabetic skin ulcers described in this study, fdECM can be a promising candidate for the treatment of other disorders, such as cardiac diseases (Fan et al., 2012; Zeng et al., 2013; Schmuck et al., 2014). Moreover, it can be used to directly reconstruct the circulatory or nervous system.

It is considered that there is potential for improvement of ECM’s efficacy. Various fibroblast types exhibit different phenotypes, protein components, secretion profiles, and mechanical properties (Savitri et al., 2020). Recent studies have demonstrated that ECMs derived from other origins likewise could enhance the function of the target tissue by promoting cell-niche interactions and tissue-specific niches (Kusuma et al., 2017; Davidov et al., 2021). Phenotype-modulation of ECM source cells also exhibited better tissue regeneration (Antich et al., 2021). Coculturing of stromal and endothelial cells also enhanced mesenchymal stem cell differentiation (Carvalho et al., 2019). Moreover, aligning human dermal fdECM for vascular graft also enhanced a-SMA and calponin expression of mesenchymal stem cells (Xing et al., 2017). These papers suggest that cells of the same origin are not necessarily needed and that ECM efficacy can be maximized through various methods. For example, if the characterization is generated dependent on the ECM extracted from different cell types, such as dermal fibroblasts or keratinocytes, or at differentiation stages, the therapeutic efficacy of ECM can be optimized. Furthermore, it is necessary to consider the change in the efficacy of ECM according to the extraction method and application platform (Zhang et al., 2022). When these efforts are implemented, cell-derived dECM can be used as a multi-functional biomaterial that can be applied to various other biomaterials, from scaffolds to hydrogels.

## Data Availability

The original contributions presented in the study are included in the article/Supplementary Material, further inquiries can be directed to the corresponding author.
