# Supplementary figures and images for: Effect of Decellularized Extracellular Matrix Bioscaffolds Derived from Fibroblasts on Skin Wound Healing and Remodeling
Source: Front Bioeng Biotechnol. 2022 Jun 29;10:865545. doi: 10.3389/fbioe.2022.865545 (PMC9277482; doi:10.3389/fbioe.2022.865545)

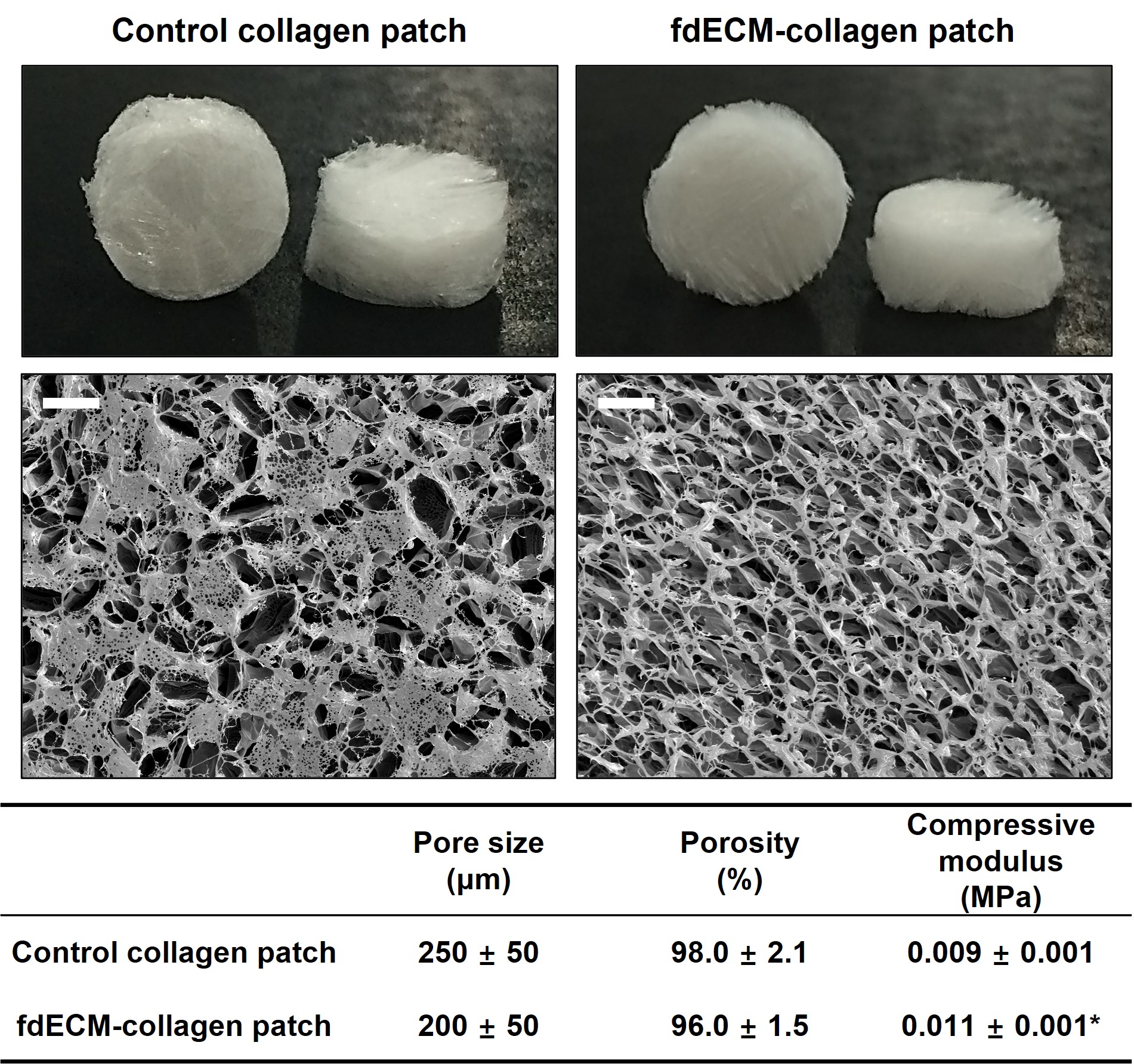

Supplement: Supplementary file 1 [file Image2.jpeg]

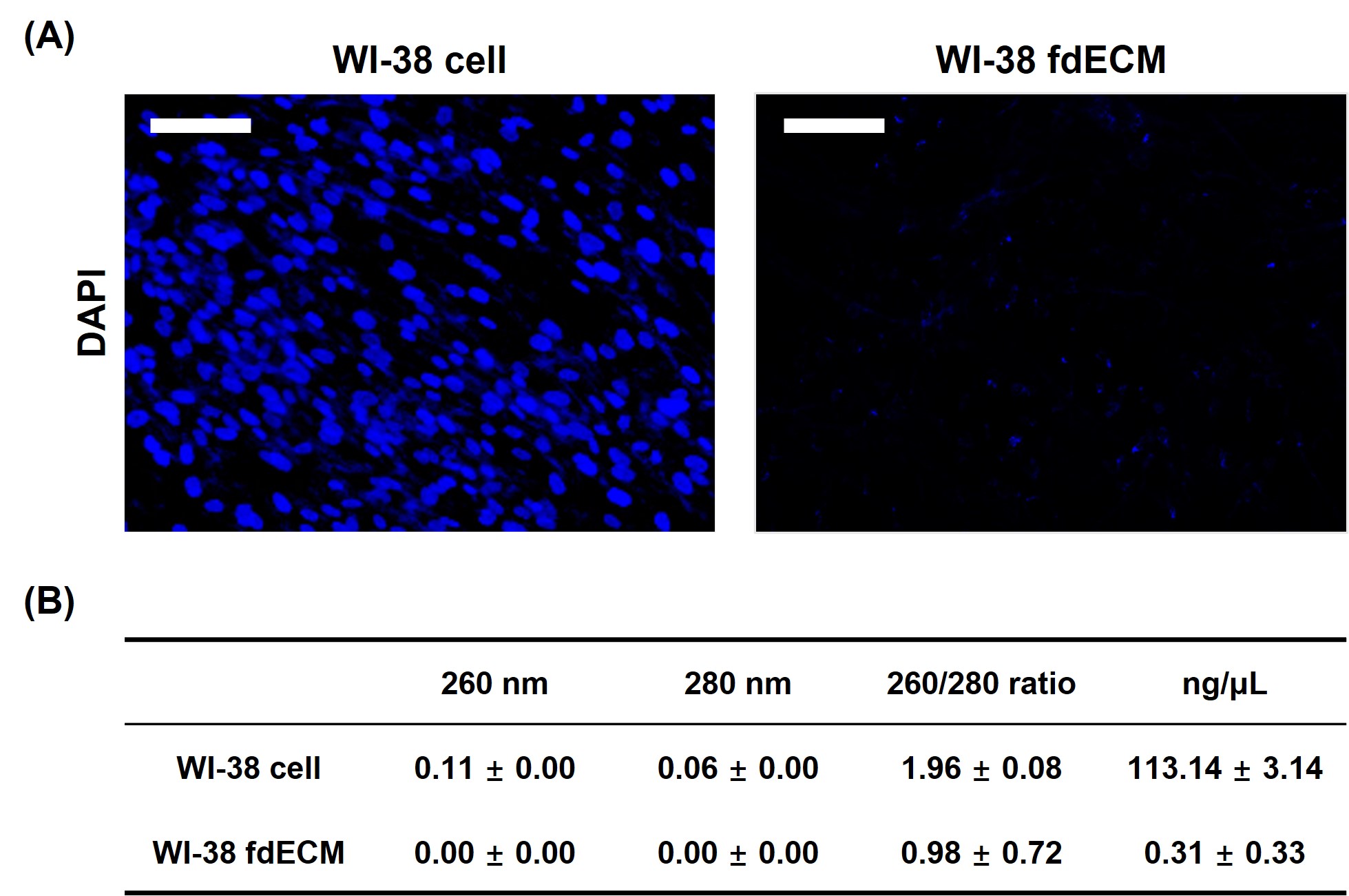

Supplement: Supplementary file 2 [file Image1.jpg]
